# CA1 hippocampal network activity changes during sleep-dependent memory consolidation

**DOI:** 10.3389/fnsys.2014.00061

**Published:** 2014-04-17

**Authors:** Nicolette Ognjanovski, Daniel Maruyama, Nora Lashner, Michal Zochowski, Sara J. Aton

**Affiliations:** ^1^Department of Molecular, Cellular and Developmental Biology, University of MichiganAnn Arbor, MI, USA; ^2^Department of Physics, University of MichiganAnn Arbor, MI, USA; ^3^Biophysics Program, University of MichiganAnn Arbor, MI, USA

**Keywords:** synaptic plasticity, fear memory, neural network, consolidation, hippocampus, extracellular recording, slow wave sleep, REM sleep

## Abstract

A period of sleep over the first few hours following single-trial contextual fear conditioning (CFC) is essential for hippocampally-mediated memory consolidation. Recent studies have uncovered intracellular mechanisms required for memory formation which are affected by post-conditioning sleep and sleep deprivation. However, almost nothing is known about the circuit-level activity changes during sleep that underlie activation of these intracellular pathways. Here we continuously recorded from the CA1 region of freely-behaving mice to characterize neuronal and network activity changes occurring during active memory consolidation. C57BL/6J mice were implanted with custom stereotrode recording arrays to monitor activity of individual CA1 neurons, local field potentials (LFPs), and electromyographic activity. Sleep architecture and state-specific CA1 activity patterns were assessed during a 24 h baseline recording period, and for 24 h following either single-trial CFC or Sham conditioning. We find that consolidation of CFC is not associated with significant sleep architecture changes, but is accompanied by long-lasting increases in CA1 neuronal firing, as well as increases in delta, theta, and gamma-frequency CA1 LFP activity. These changes occurred in both sleep and wakefulness, and may drive synaptic plasticity within the hippocampus during memory formation. We also find that functional connectivity within the CA1 network, assessed through functional clustering algorithm (FCA) analysis of spike timing relationships among recorded neurons, becomes more stable during consolidation of CFC. This increase in network stability was not present following Sham conditioning, was most evident during post-CFC slow wave sleep (SWS), and was negligible during post-CFC wakefulness. Thus in the interval between encoding and recall, SWS may stabilize the hippocampal contextual fear memory (CFM) trace by promoting CA1 network stability.

## Introduction

Sleep plays an essential role in promoting various forms of memory consolidation (Aton et al., 2009b) and plasticity in brain circuits *in vivo* (Aton et al., 2009a, 2013, 2014; Seibt et al., 2012). Recent work has taken advantage of single-trial training paradigms to assess sleep effects on memory processes dependent on circuit plasticity (Aton et al., 2014). An example is single-trial contextual fear conditioning (CFC) in mice (placement in a novel context, followed by foot shock). Such conditioning results in a long-lasting fearful memory, measured as contextual freezing behavior upon return to the CFC context. In this paradigm, sleep within the first 5 h following CFC is an absolute requirement for long-term contextual fear memory (CFM) consolidation (Graves et al., 2003; McDermott et al., 2003; Vecsey et al., 2009; Prince et al., 2014).

Recent work has been aimed at understanding the relationship between sleep and the intracellular events required for both synaptic plasticity and memory formation. The cellular mechanisms underlying CFM *in vivo* are also critical for long-term potentiation (LTP) of CA1 hippocampal synapses *in vitro*. CFM consolidation requires both CA1 network activity (Daumas et al., 2005), and the activation of kinase and protein synthesis pathways (Bourtchouladze et al., 1998; Lattal and Abel, 2004; Sindreu et al., 2007), in the hours immediately following CFC. Because these pathways are also required for CA1 LTP, one possibility is that sleep interferes with CFM consolidation by disrupting synaptic potentiation in CA1. Recent studies have clarified intracellular events in the hippocampus during sleep vs. sleep loss (Vecsey et al., 2012) and have defined how intracellular signaling pathways are altered by sleep deprivation to impair CFM (Vecsey et al., 2009). The pathways disrupted in the hippocampus by sleep loss (e.g., mTOR-mediated activation of protein synthesis, kinase-mediated protein phosphorylation) are essential for CA1 LTP (Vecsey et al., 2009, 2012), and critically, sleep deprivation itself interferes with CA1 LTP (McDermott et al., 2003; Kopp et al., 2006).

In contrast to our increasing understanding of the cellular and molecular effects of sleep and sleep deprivation in the hippocampus, almost nothing is known about how the hippocampal network activity changes unique to sleep contribute to memory consolidation. Two hippocampal network oscillations—theta (4–12 Hz) and sharp-wave/ripple (150–200 Hz) events—are hypothesized to promote episodic memory consolidation (Wetzel et al., 1977; Eschenko and Sara, 2008; Girardeau et al., 2009; Popa et al., 2010). These oscillations occur most prominently during rapid eye movement (REM) sleep and slow wave sleep (SWS), respectively. However, very little is known about the role of such oscillations in sleep-dependent memory consolidation. Even less is known about whether activity changes among individual hippocampal neurons occur during memory consolidation. Recent *in vitro* studies suggest that membrane excitability increases in CA1 neurons follow initial learning (McKay et al., 2009, 2013), and that long-term (i.e., 72 h) sleep deprivation reduces membrane excitability (McDermott et al., 2003). However, it remains unclear how such changes are expressed *in vivo*, or what role sleep might play in regulating *in vivo* activity changes.

To clarify how hippocampal network activity changes *in vivo* during active memory consolidation, we carried out continuous stereotrode recording of CA1 neuronal firing and local field potential (LFP) activity in mice. Recordings spanned a 24 h baseline period and for 24 h following either single-trial CFC or Sham conditioning (placement in a novel context without associated foot shock; a control for behavioral procedures not associated with CFM). We assessed how neuronal and network activity in CA1 was altered as a function of behavioral state and active CFM consolidation. Specifically, we quantified state-specific changes in CA1 neurons’ firing rates and power spectral density in CA1 LFPs following either CFC or Sham conditioning. We also assessed changes in the temporal dynamics of functional communication between CA1 neurons in the hippocampal network as a function of CFC and behavioral state. By clarifying the network-level activity changes associated with consolidation of CFM, we hope to shed light on other CA1-mediated functions, such as object recognition memory (Clarke et al., 2010), spatial contextual memory (Dupret et al., 2010; Barbosa et al., 2012), and spatial representation (Henriksen et al., 2010).

## Materials and methods

### Mouse handling, surgical procedures, and driveable headstage placement

All animal husbandry and surgical/experimental procedures were approved by the University of Michigan UCUCA board for animal care and use. Throughout all experimental procedures, mice were kept on a 12 h:12 h light:dark cycle (lights on at 8 AM), and were given food and water *ad lib*.

At age 2–6 months, male C57BL/6J mice (Jackson) were implanted with custom-built, driveable headstages under isoflurane anesthesia, using previously-described techniques (Aton et al., 2013, 2014). Each driveable headstage was composed of two bundles (each approximately 200 μm in diameter, spaced 1–1.5 mm apart) of 7 stereotrodes each (25 μm nichrome wire, California Fine Wire) wired onto Neuralynx electrode interface boards (EIB-36, Neuralynx). During surgical placement, stereotrode bundles were placed within right-hemisphere CA1. Reference and ground electrodes (silver-plated copper wire, Alpha Wire) were placed over left-hemisphere hippocampus and cortex, and 3 EMG electrodes were placed deep in the nuchal muscle.

### Recording procedures

Chronic stereotrode recording was carried out using general procedures described previously (Aton et al., 2013, 2014). After 1 week of postoperative recovery, mice housed in their home cages were placed within a sound-attenuated sleep-recording chamber (Med Associates), and headstages were connected to a lightweight cable to record neural signals. Over a 3–5 day period, mice were habituated to the recording chamber and were handled daily for at least 10 min. During this time, stereotrode bundles were slowly advanced into the hippocampus in 10–20 μm steps, until stable recordings were obtained (indicated by continuous presence of spike waveforms on channels for at least 24 h). Following this period of habituation and electrode advancement, all experiments began with a 24 h baseline recording period, starting at lights-on. Signals from each electrode were split and differentially filtered to obtain spike data (200 Hz–8 kHz) and local field potential/electroencephalographic data (LFP/EEG; 0.5 Hz–200 Hz) at each recording site. Data were amplified at 20×, digitized, further digitally amplified at 20–100×, and recorded using Plexon Omniplex hardware and software (Plexon Inc.; Dallas, TX).

### Lesioning and laminar analysis of recording sites

At the end of experimental recording procedures, mice were anesthetized with isoflurane and all electrode sites were lesioned (2 mA, 3 s per wire), after which mice were euthanized and perfused with formalin. To verify CA1 electrode placement, the hippocampus was post-fixed and sectioned at 50 μm for cresyl violet staining and reconstruction of stereotrode bundle tracts and recording site lesions, using previously described procedures (Aton et al., 2013, 2014).

### CFC

Following 24 h baseline recording (within 1 h of lights-on), mice underwent either CFC (Graves et al., 2003) or Sham conditioning (*n* = 6 per group). Mice were placed in a novel conditioning chamber with walls made of clear Plexiglas and a shock grid floor. Chamber walls and floor were cleaned with 70% ethanol both prior to and immediately following conditioning. Mice were placed into the chamber and allowed to explore freely for either 150 s (CFC mice) or 180 s (Sham mice). CFC mice then received a 2 s foot shock (0.75 mA; administered via a Med Associates Aversive Stimulator/Scrambler), and were left in the conditioning chamber for an additional 28 s. Throughout these procedures, mice were continuously video monitored using Plexon Cineplex software. Following conditioning, mice were returned to their home cage in the sleep-recording chamber and underwent an additional 24 h period of undisturbed recording prior to contextual fear behavioral assessment.

**Figure 1:**
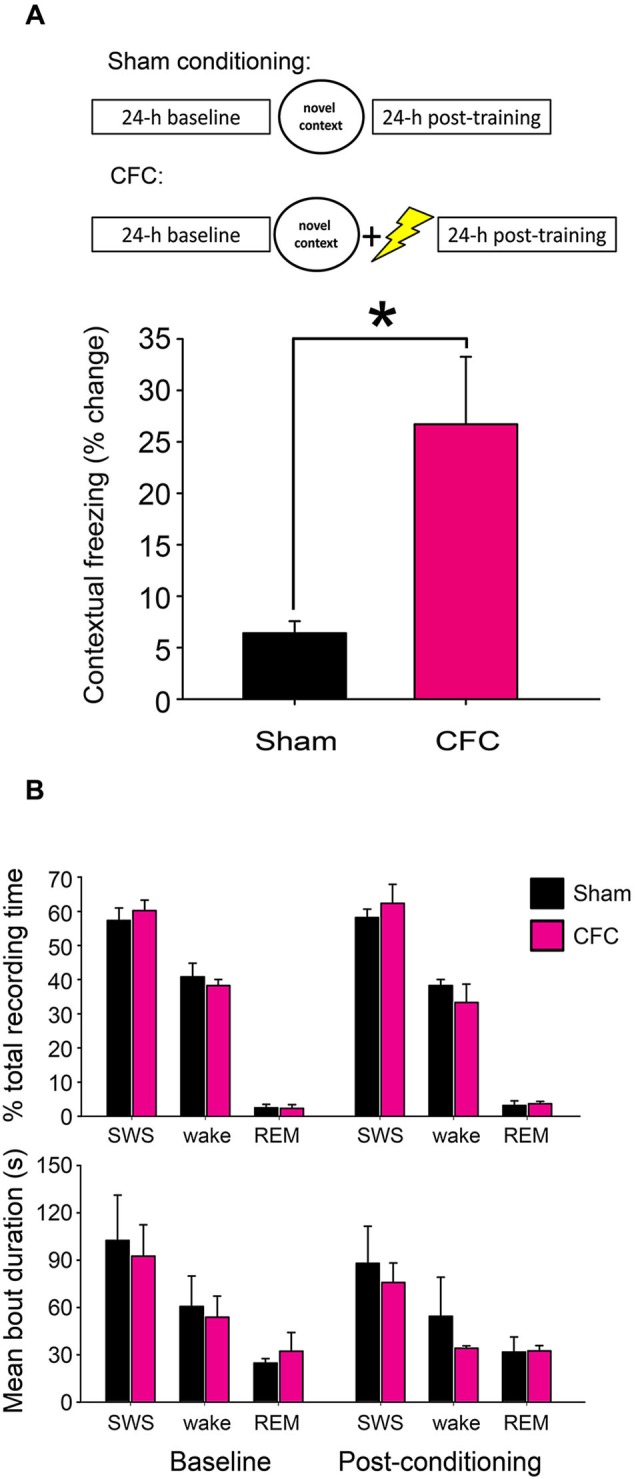
**CFC initiates fear memory formation without significantly altering sleep-wake behavior. (A)** Experimental overview and quantification of context-specific freezing in Sham and CFC mice (*n* = 6 per group). * indicates *p* < 0.05, Student’s *t*-test. **(B)** Sleep architecture in the 24 h following CFC was not significantly altered, either relative to pre-CFC baseline, or relative to the 24 h following Sham conditioning. Effects of group (Sham vs. CFC) and time relative to training (baseline vs. post-conditioning) on % total recording time or bout duration for each state, *N.S*., two-way RM ANOVA.

Twenty-four hour following contextual fear or sham conditioning, mice were returned to the conditioning chamber for 5 min, during which behavior was continuously video monitored. Freezing behavior (and CFM) was subsequently assessed from video recordings using previously-described methods (Vecsey et al., 2009). To quantify CFM in each mouse, context-specific freezing was quantified as a change in the percentage of total recording time spent in stereotyped freezing behavior between the 5 min test period and the pre-shock interval in the initial training period (i.e., % time spent freezing at test—% time spent freezing at baseline; Figure 1A).

### Sleep/wake, firing rate, and LFP analysis

Intrahippocampal LFP and nuchal EMG signals were used to assign polysomnographic data into periods of REM sleep, SWS, and waking states over 10 s intervals using custom software. The proportion of time spent in REM, SWS, and waking (and mean bout duration for each state) was calculated during the baseline and post-conditioning recording periods using standard conventions (Aton et al., 2013).

**Figure 2:**
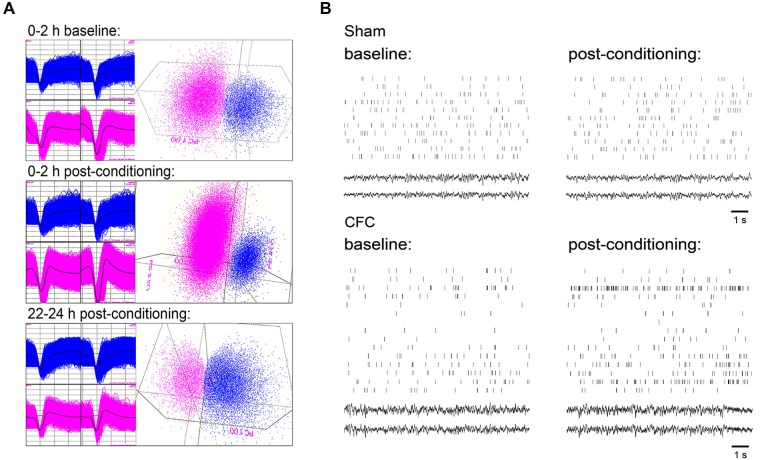
**CA1 neuronal spiking and field potential recordings. (A)** Representative spike waveforms for two CA1 neurons which were continuously recorded on a single stereotrode and reliably discriminated across 24 h baseline and 24 h post-CFC periods (left), and mapping of spike waveform clusters in three-dimensional principal component space (right). Data shown comprise waveforms collected over 2 h windows, either 24 h prior to CFC (top), immediately following CFC (middle), or immediately prior to contextual fear assessment (bottom). **(B)** 10 s rasters of firing for all CA1 neurons stably recorded from a representative mouse in Sham and CFC conditions (and corresponding LFPs). Rasters are taken from intervals of SWS within the first hour of both the baseline and post-conditioning periods.

Single-neuron data were discriminated offline using standard principle-component based procedures (Offline Sorter; Plexon). Individual neurons were tracked throughout each experiment on the basis of spike waveform, relative spike amplitude on the two stereotrode recording wires, relative positioning of spike waveform clusters in three-dimensional principal component space, and neuronal subclass (e.g., FS vs. principal) (Aton et al., 2013, 2014). Only those neurons that were verifiably discriminated and continuously recorded throughout each experiment (i.e., those that were stably recorded across 24 h baseline and 24 h post-conditioning recording) were included in analyses of ongoing network activity. An example of representative spike data from two neurons recorded on a single stereotrode is shown in Figure 2A at different timepoints across baseline and post-conditioning recording periods.

**Figure 3:**
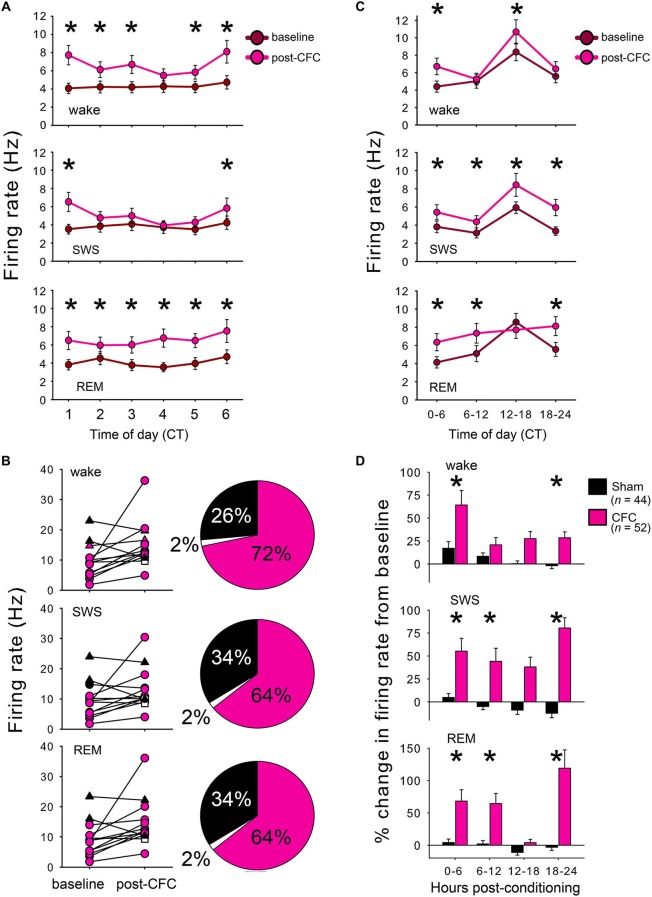
**CFC induces long-lasting increases in CA1 neuronal firing rates. (A)** Mean firing rates for CA1 neurons increased within the first hours following CFC. For all 3 states, main effect of conditioning *p* < 0.05; conditioning × time interaction *p* < 0.001 for wake and SWS, two-way RM ANOVA. **(B)** Firing rate changes in each state are shown for individual neurons within a single representative CFC mouse (left) and the proportion of neurons showing increases, decreases, or no change in firing after CFC are summarized across all experiments (right). Neurons with a >5% increase in firing from baseline are shown in pink, those with >5% decrease are shown in black, and those with no change are shown in white. **(C)** CA1 neuronal firing remained elevated across the 24 h post-CFC recording period. For all 3 states, main effect of conditioning *p* < 0.05; conditioning × time-of-day interaction *p* < 0.005, two-way RM ANOVA. **(D)** CFC and Sham conditioning differentially affected firing rate. Post-conditioning firing rates for each neuron were expressed as a % change from the corresponding period of baseline recording. Effects of group (Sham vs. CFC) and time relative to training (baseline vs. post-conditioning) *p* < 0.001 for each state, two-way RM ANOVA. * indicates *p* < 0.05, Holm-Sidak *post hoc* test for baseline vs. post-CFC comparisons.

For each reliably-discriminated neuron (*n* = 44 for Sham conditioned mice, *n* = 52 for CFC mice), mean firing rates were calculated separately within each behavioral state (REM, SWS, and wakefulness) across the 24 h baseline period and 24 h post-conditioning period. Mean firing rates in each state were then calculated in 1 h and 6 h windows across each recording period, as shown in Figures 3A and 3C, respectively. Firing rate changes after conditioning were then expressed for each neuron as a % change from baseline in each 6 h window, as shown in Figure 3D. For analysis of direction of firing rate changes among individual neurons (recorded within an individual experiment, or across all experiments (left and right, respectively, Figure 3B)), increases and decreases in firing rate were estimated as those changes >5% from baseline. Neurons expressing an increase or decrease of ≤5% from baseline were categorized as having no change in firing rate.

**Figure 4:**
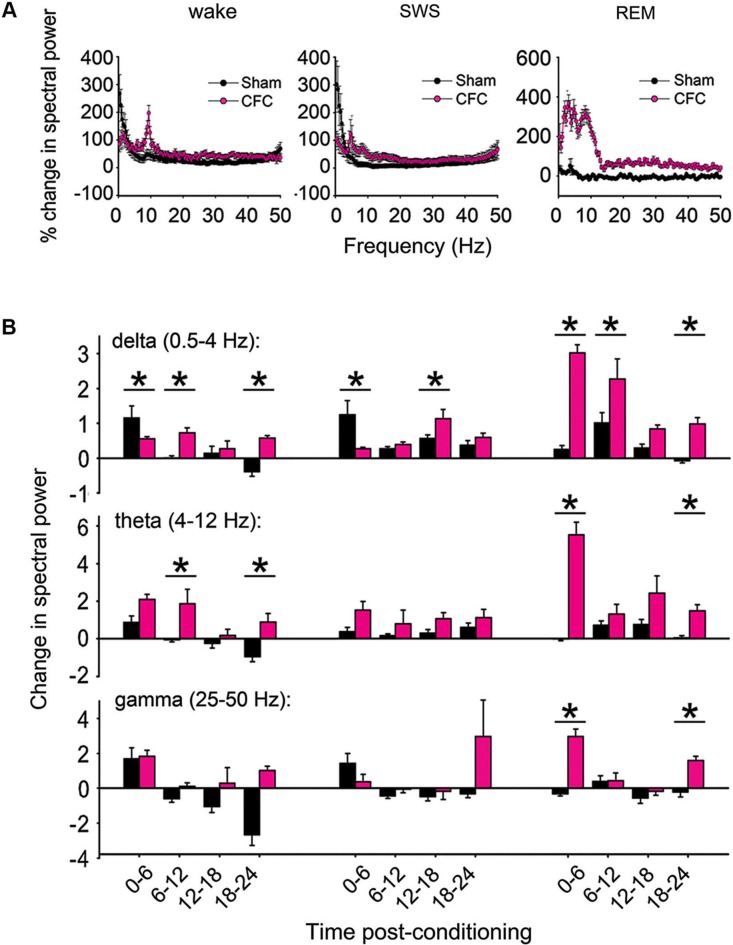
**CFC induces long-lasting increases in CA1 LFP oscillatory activity. (A)** Changes in spectral power (from baseline to post-conditioning recording periods) are shown from 0–50 Hz over the first 6 h post-conditioning in wakefulness, SWS, and REM. **(B)** % changes in spectral power were summed across delta, theta, and gamma-frequency bands to compare band-specific changes following consolidation. Values represent the arithmetic sum of these changes (measured at 0.4 Hz intervals) × 10^3^. While the largest post-CFC changes were seen in the theta-frequency range during REM (with relative increases peaking at around 7–4 Hz), significant increases in theta were also present following CFC in wakefulness, as in delta and (to a lesser extent) gamma-frequency bands. * indicates *p* < 0.05, Holm-Sidak *post hoc* test for baseline vs. post-CFC comparisons.

Raw LFP power was calculated on each channel where spike data was stably recorded throughout the experiment. Power spectral density (PSD) values were quantified in 0.4 Hz bands, and average power in each band was assessed separately within each behavioral state (REM, SWS, and wakefulness) across 6 h windows. Changes in power at each frequency band were quantified from raw LFP traces as a % change from baseline within each window (as shown in Figure 4A). For quantitative analysis of LFP changes, changes from baseline were summed across the following frequency bands for each mouse: delta (0.5–4 Hz), theta (4–12 Hz (and for comparison, also in a narrower 4–7 Hz band)), and gamma (25–50 Hz). Values shown in Figure 4B represent the arithmetic sum of % changes for each 0.4 Hz frequency measure across each respective band, × 10^3^.

### Functional clustering algorithm and network stability analysis

**Figure 5:**
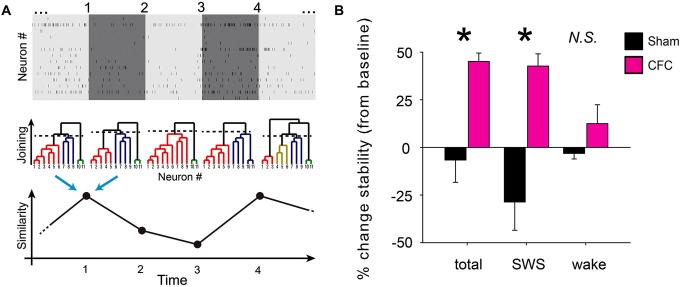
**CA1 network communication becomes more stable during SWS following CFC. (A)** Schematic depiction of functional clustering algorithm (FCA) and subsequent network stability analysis. Recorded spike trains are divided into adjoining 1 min intervals (top). FCA is performed on every 1 min interval (middle) to assess functional connectivity among the recorded neurons. This analysis yields a dendrogram of network functional structure at each interval. The stability of network connectivity is established from the evaluation of the similarity of neuronal functional clustering between consecutive intervals (bottom). **(B)** Minute-to-minute network stability was averaged across baseline to post-conditioning periods. Post-conditioning changes in average stability (from baseline) are shown for Sham and CFC mice. While significant differences in stability changes were seen between the two groups when analysis was carried out across all states (total) or across intervals occurring in SWS, no significant stability differences seen for analysis over intervals of wakefulness (wake). * indicates *p* < 0.01 and *p* < 0.005 respectively for all-state and SWS-specific analysis, Student’s *t*-test.

To assess dynamic network reorganization in the hippocampus following conditioning, and as a function of behavioral state, we used a functional clustering algorithm (FCA; Feldt et al., 2009) to assess functional network structure based on firing patterns among CA1 neurons. The FCA is a reduction-based algorithm, in which pairwise correlations between neurons in a network are calculated and the most strongly correlated pairs are iteratively merged together into a single spike train. Those trains consisting of progressively merged cells’ activity are progressively joined, forming clusters of cells exhibiting similar spatio-temporal activity patterns over time. The clustering stops when the most highly correlated pair among the remaining spike trains no longer exceeds a set threshold. These correlations were calculated based on the average minimum time-distance (AMD) between the spikes of the two trains (with shorter times indicating greater correlation), normalized with reference to uncorrelated (Poisson) spike trains with the same firing rate. This normalization removes the frequency-dependence of the average minimum time metric and allows comparisons to be made between cells with distinct firing rates. FCA combined with the normalized AMD measure was used to identify number of functional clusters and identities of neurons belonging to given cluster. These interdependencies characterize the functional connectivity structure among recorded CA1 neurons and are depicted as dendrograms for given time interval (Figure 5A). The data was divided into 1 min bins and dendrograms were generated for the entire population of stably recorded CA1 neurons across each 1 min recording interval. Dendrogram joining values were assigned to each pair. The joining values were defined as Jij=−log(AMDijPij), where AMD_ij_ is the average minimal distance between *i*-th and *j*-th train and P_ij_ its theoretical value obtained from Poisson spike distributions having equivalent numbers of spikes. Based on these dendrograms, we established a connectivity vector for every neuron.

To assess whether CFC resulted in a lasting, stable change in CA1 functional connectivity, we next compared the connectivity matrices from adjacent 1 min time intervals using cosine similarity. This similarity analysis yielded values between −1 and 1, indicating the level of similarity between dendrogram structures from adjacent 1 min recording intervals, with a value of 1 indicating that the functional connectivity of the cells did not change in any way (i.e., the cells in the two time intervals formed exactly the same functional clusters with exactly the same joining distances). Thus the metric returns the degree of similarity between two adjacent dendrograms, but does not elucidate the scope of their changes (e.g., changes in the number of clusters, neuronal identities assigned to a given cluster, joining distances, etc.). The resulting minute-to-minute similarity values were averaged over the entire 24 h duration of baseline and post-conditioning recording. Changes in average stability between the baseline and post-conditioning periods were calculated separately using data from: (1) all behavioral states; (2) from epochs of SWS only; or (3) from epochs of wakefulness only (Figure 5B). Due to the relative infrequency and short duration of REM epochs (which typically lasted less than 1 min each) there was an insufficient number of successive recording epochs to reliably calculate network stability changes specifically within REM epochs.

## Results

### CFC induces CFM without altering sleep behavior

CFM was assessed using previously described methods (Vecsey et al., 2009). As anticipated, mice receiving a foot shock in the context of exploring a novel test chamber (CFC) showed significantly increased freezing behavior when returned to the same environment 24 h later (*p* < 0.05 for CFC vs. Sham, Student’s *t*-test; Figure 1A). While CFM consolidation is known to require sleep behavior, neither CFC nor Sham conditioning caused significant changes in subsequent sleep architecture (i.e., % of time spent in SWS, REM, or wakefulness, or mean duration of SWS, REM or wakefulness bouts) from the baseline recording period (Figure 1B). Two-way repeated-measures ANOVA found no significant effect of either group (Sham vs. CFC) or time relative to training (baseline vs. post-conditioning) on sleep architecture measures. Thus the role of sleep in promoting CFM consolidation is not associated with either overall increases in sleep time, alterations in SWS: REM sleep time ratio, or greater sleep continuity following CFC.

### CFM consolidation is associated with increased CA1 neuronal activity

To assess changes in neuronal activity associated with CFM consolidation, firing patterns of individual CA1 hippocampal neurons were tracked continuously over the 24 h baseline recording period and for 24 h following conditioning. Firing rate changes associated with Sham conditioning and CFC were assessed among those neurons stably recorded across the entire baseline and post-conditioning periods (from which representative example data is shown in Figures 2A–B). CA1 firing rates for CFC mice increased significantly in all behavioral states within the first hours following CFC (Figure 3A; main effect of conditioning *p* < 0.005, *p* < 0.001, and *p* < 0.05 for wake, REM, and SWS, respectively; conditioning × time interaction *p* < 0.001, *N.S*., and *p* < 0.001 for wake, REM, and SWS, respectively, two-way RM ANOVA). These early post-CFC firing rate changes were not uniform across the population of CA1 neurons. While the majority of neurons recorded from CFC mice (>60%) showed firing rate increases across all states, 25–35% showed decreases, and a much smaller minority (<5% of neurons from all recordings) showed no change (less than 5% increase or decrease from baseline; Figure 3B). Post-CFC firing rate changes were maintained across the 24 h period following CFC (Figure 3C; main effect of conditioning *p* < 0.05, *p* < 0.01, and *p* < 0.005 for wake, REM, and SWS, respectively; conditioning × time-of-day interaction *p* < 0.005, *p* < 0.001, and *p* < 0.005 for wake, REM, and SWS, respectively, two-way RM ANOVA). In contrast, firing rates for Sham mice did not change significantly during REM or wakefulness after Sham conditioning (main effect of conditioning *N.S*., two-way RM ANOVA), and showed a slight but significant decrease after Sham conditioning within SWS (*p* < 0.05, two-way RM ANOVA). When firing rate changes post-conditioning were expressed as a percent change from baseline, significant differences between Sham and CFC were evident, with mean increases of >50% present in all states immediately after conditioning (Figure 3D). The tendency for increased firing among CA1 neurons after CFC was present throughout the entire 24 h post-conditioning recording period. Thus the duration of firing rate changes we see *in vivo* (lasting at least 24 h following conditioning) is consistent with the reported timecourse of CA1 neurons’ intrinsic excitability changes observed *in vitro* after classical conditioning in various rodent models (Moyer et al., 1996; McKay et al., 2009, 2013).

### CA1 field activity is altered during CFM consolidation

To determine how hippocampal network activity patterns are affected during CFM consolidation, raw spectral power for CA1 LFPs were compared in each behavioral state between corresponding 6 h time windows in the baseline and post-conditioning periods (Figure 4A). Comparisons of percent changes in LFP power across frequency bands showed clear differences between CFC and Sham mice. To characterize these differences, power spectral changes from baseline were summed across delta (0.5–4 Hz), theta (4–12 Hz), and gamma (25–50 Hz) frequency bands. In the first 6 h post-conditioning, CFC showed smaller increases in delta-activity than Sham mice during both wakefulness and SWS, and larger increases in REM (Figures 4A–B). At subsequent time points, delta-power showed larger increases in CFC mice than Sham mice—an effect that was present across all states (main effect of conditioning *p* < 0.01, *p* < 0.001, and *N.S*. for wake, REM, and SWS, respectively; conditioning × time-of-day interaction *p* < 0.001 for all states, two-way RM ANOVA). Theta-frequency activity showed even larger increases following CFC, which were present in REM and wakefulness (but not SWS) throughout the post-CFC recording period (main effect of conditioning *p* < 0.005, *p* < 0.001, and *N.S*. for wake, REM, and SWS, respectively; conditioning × time-of-day interaction *p* < 0.005 for REM, *N.S*. for wake and SWS, two-way RM ANOVA). Because the range of theta-frequencies reported here is broader than that published in some studies (which can be restricted to a range as narrow as 4–7 Hz), we also compared changes in the 4–7 Hz band, with nearly identical results (main effect of conditioning *p* < 0.001, *p* < 0.001, and *N.S*. for wake, REM, and SWS, respectively; conditioning × time-of-day interaction *p* < 0.001, *p* < 0.05, and *N.S*. for wake, REM, and SWS, respectively, two-way RM ANOVA). Slight increases in gamma were also present following CFC, but these changes were restricted to REM (main effect of conditioning *p* < 0.001; conditioning × time-of-day interaction *p* < 0.001, two-way RM ANOVA).

To test whether state-specific changes in activity were proportional to firing rate changes following CFC, we compared firing rate and LFP power changes across individual CA1 recording sites in the first 6 h post-conditioning (a time point when most of these changes are either maximal, or near-maximal). For all states and all frequency bands quantified, there was no correlation between changes in LFP power and changes in firing rate (*p* > 0.1 for all measures, Pearson correlation).

### CA1 network structure is stabilized during CFM consolidation

Recent quantitative analysis of hippocampal immediate-early gene expression has indicated that activity across the network of CA1 neurons during rest reflects the specific network activation patterns generated in prior waking experience (Marrone et al., 2008). This suggests that a memory trace, or engram, may be continuously present in CA1 at the level of network activation (and perhaps network functional connectivity) long after a learning experience ends. To test whether this is true of the CA1 network following CFC, we used a functional clustering algorithm (FCA; Feldt et al., 2009) to quantify functional connectivity between CA1 neurons. We also used minute-to-minute comparisons of CA1 network functional connectivity to quantify the stability of network activity patterns at baseline and after conditioning.

Stability of network functional structure was assessed over time by comparing FCA-generated network architecture between successive 1 min intervals across the entire baseline and post-conditioning periods (Figure 5A). The term “stability” in this case means the similarity (in time) of functional connectivity among recorded neurons. In this sense, more stable networks maintain similar functional clusters over time (i.e., the same neurons remain within given functional clusters, and neither the number of clusters nor the joining distance change significantly). Using this metric, average minute-to-minute stability values were calculated across each recording period, and changes in average stability across the recorded CA1 network post-conditioning were expressed as a percent change from baseline in each mouse. CA1 network structure stability increased significantly after conditioning in CFC mice, but not in Sham mice (Figure 5B). This stability increase was evident when all successive 1 min intervals were included in stability analysis, regardless of behavioral state, and also when only intervals spent in SWS were analyzed separately (*p* < 0.01 and *p* < 0.005 respectively for all-state and SWS-specific analysis, Student’s *t*-test). However, the same stability increase was not seen across post-CFC intervals of wakefulness (stability changes for CFC vs. Sham *N.S*., Student’s *t*-test). Stability across REM intervals could not be separately assessed due to the relatively brevity and infrequency of REM episodes, which reduced the reliability of stability measurements.

## Discussion

These studies were aimed at assessing sleep-associated changes in CA1 network activity that might contribute to the sleep-dependence of CFM consolidation. We found that single-trial CFC induces CFM without altering sleep architecture over the 24 h CFM consolidation period. CFC does, however, lead to three long-lasting changes in either the activity of individual CA1 neurons or the interactions of these neurons within the hippocampal network. These changes are discussed in detail below:

### Post-CFC firing rate increases in CA1 neurons

First, we find that neuronal firing rates increase immediately after CFC *in vivo*, and remain elevated over the course of 24 h of post-CFC recording. This increase was specific to CFC, as similar firing rate changes were not seen after Sham conditioning, where exploration of a novel context is not paired with foot shock. Critically, the time course of these changes is similar to that of excitability changes measured *in vitro* in mouse, rat, and rabbit CA1 neurons following aversive conditioning (Moyer et al., 1996; McKay et al., 2009, 2013).

Recent studies of the underlying mechanisms for these changes have shown that aversive conditioning leads to decreased expression of KCNN2, an apamin-sensitive SK channel, in the hippocampus (McKay et al., 2012). Activation of SK channels blocks conditioning-induced excitability changes in both CA1 pyramidal neurons and interneurons, and impairs learning (McKay et al., 2012, 2013). Because SK channels play a critical role in regulating after hyperpolarizing (AHP) currents and thus firing rates in CA1 (Pedarzani et al., 2005), excitability changes associated with SK channel reductions may mediate the firing rate increases we see *in vivo* after CFC. Critically, sleep may play an important role in maintaining conditioning-induced reductions in SK channel expression. A recent study examining sleep- and sleep deprivation-mediated gene expression changes found that expression of KCNN2 is reduced during sleep and increased during sleep deprivation in multiple brain areas (Mackiewicz et al., 2007). Such a mechanism may explain the effect of sleep loss in reducing membrane excitability in CA1 (McDermott et al., 2003).

What effect do firing rate increases have on the hippocampal network during CFM consolidation? While CA1 network activity in the hours following CFC is essential for CFM (Daumas et al., 2005), the role of increased activity in the consolidation process is unknown. One possibility is that increased firing rates among CA1 neurons drives synaptic plasticity in the network throughout the post-CFC consolidation window. Firing rate contributes to the sign (LTP or LTD) of spike-timing-dependent plasticity (Feldman, 2012), and for some CA1 synapses, firing rate appears to be more important than pre-vs.-postsynaptic spike timing for determining the sign of plasticity (Wittenberg and Wang, 2006). Based on available data from *in vitro* studies, it seems plausible that the increase in firing we observe after CFC *in vivo* biases CA1 neurons toward synaptic potentiation, which appears to be essential for multiple forms of CA1-dependent memory. Interference with cellular pathways required for synaptic potentiation in the hours following CFC (through either drug treatments, or behaviorally through sleep deprivation) impairs CFM consolidation (Bourtchouladze et al., 1998; Vecsey et al., 2009), and postsynaptic potentiation has been measured within CA1 *in vivo* within 6 h following object recognition training (Clarke et al., 2010). Because synaptic potentiation clearly plays an important role in long-term memory formation, it stands to reason that enhanced neuronal firing could promote consolidation through this mechanism.

### Post-CFC increases in network oscillations

Second, we find that LFP oscillations in CA1 are specifically increased in multiple frequency bands following CFC (relative to Sham conditioning), with a time course similar to that seen for firing rate changes (i.e., lasting up to 24 h). While relatively modest changes were seen for gamma-oscillations, which were significantly increased following CFC only during REM sleep. In terms of magnitude, the largest changes in LFP activity were increases in the theta (4–12 Hz) range, particularly during REM sleep. CA1 neurons show natural oscillations in membrane potential *in vitro* that resonate with exogenous input at theta-frequencies (Leung and Yu, 1998), and CA1 theta-oscillations *in vivo* are specifically augmented during novel experiences which engage the hippocampus (Penley et al., 2013). Because theta-frequency stimulation can induce LTP in CA1 *in vitro* (Woo et al., 2000), and driving theta-oscillations in the hippocampus *in vivo* promotes memory consolidation (Wetzel et al., 1977), it is tempting to speculate that these oscillations drive the network plasticity underlying memory formation. Our data suggest that naturally-occurring enhancements in theta-frequency CA1 oscillations accompany CFM consolidation. These increases are most prominent following CFC in REM sleep (a state in which prominent hippocampal theta-oscillations are a consistent feature), but are also clearly present in wakefulness. These findings raise two questions. First, what network changes underlie these long-term increases in theta-frequency activity? One possibility is that CFC leads to rapid alterations in hippocampal inputs (e.g., input from the medial septum) which could subsequently drive theta-activity more robustly in CA1 (Hasselmo, 2005). Another is that CFC alters intra-hippocampal network connectivity, which could lead to more coherent theta-oscillatory activity. While our current data do not address the former, our analyses of CA1 network structure (see below) suggest that the latter possibility is plausible. A second question is whether, and how, these oscillations contribute to memory consolidation. As is true for increases in neuronal firing, it seems plausible that enhancing theta-oscillations could promote consolidation by creating optimal conditions for synaptic potentiation within hippocampal circuits. Moreover, because increased theta-frequency coherence between the hippocampus and other brain areas (e.g., the amygdala and prefrontal cortex) is specifically associated with other forms of memory consolidation (Benchenane et al., 2010; Popa et al., 2010), it is also possible that enhanced CA1 theta-rhythms could drive systems-level memory consolidation during sleep (Aton et al., 2009b).

### Post-CFC stabilization of the CA1 network

The third change we observe in CA1 after CFC is a significant stabilization of functional connectivity within the CA1 network. Network stability increases after conditioning were not seen in any of the Sham mice, but were present in all CFC mice. Importantly, and in contrast to the firing rate and LFP changes we see following CFC, significant increases in network stability after CFC were *not* seen during periods of wakefulness, but were clearly seen in SWS. Thus increased network stability is one feature of network activity that is associated specifically with sleep (and not associated with wakefulness) during CFM consolidation. Because sleep is necessary for long-term memory formation in this system, it stands to reason that network-level changes associated with sleep are critical for the consolidation process. Stabilization of spike-timing relationships within CA1 may be a plausible strategy for preserving a memory trace (i.e., an engram) of CFC during consolidation. In this case, the memory trace would be weakened by replacing sleep with wakefulness (i.e., with sleep deprivation)—which could provide a network-level mechanistic explanation for why sleep deprivation disrupts mnemonic function.

“Replay” of experience-associated sequences of network activity during subsequent rest has been hypothesized to play an important role in memory consolidation (Abel et al., 2013). However, most studies of replay involve recording activity patterns from animals following repetition of a relatively familiar task; i.e., the animals under study have been trained over a period of days to weeks prior to recording. Thus the replay described in these studies is not temporally associated with consolidation of new memories; it might more fairly be described as occurring following practice of a very familiar task. In almost no case has sequential pattern reactivation been demonstrated in the context of active consolidation of memory following a novel learning experience. While the stability measure reported here does not necessarily require *sequential* reactivation of specific neuronal firing patterns, it does quantify the degree to which the pattern of functional connectivity (based on relative spike timing) among recorded neurons remains stable over time. Critically, the increase in stability we see following CFC is maintained across the entire 24 h post-CFC period, while sequential replay is typically reported for only a few minutes following experience (Aton et al., 2009b). Thus the longer-term stabilization of network activity patterns we see following CFC may be a true network-level correlate of *de novo* memory consolidation. The fact that increased stability is associated specifically with a behavioral state required for consolidation suggests that it may play a functional role in protecting memory traces at their most labile state, across the first 24 h following encoding.

Taken together, the neuronal and network activity changes we have found comprise one of the first descriptions of *in vivo* electrophysiological changes in CA1 corresponding to active fear memory consolidation. Future studies will be aimed at better understanding whether similar network level changes occur following learning in other brain structures, the role of sleep in promoting these changes, as well as which network-level changes are necessary, and sufficient, for memory consolidation.

## Conflict of interest statement

The authors declare that the research was conducted in the absence of any commercial or financial relationships that could be construed as a potential conflict of interest.
